# Giant submandibular sialolipoma masquerading as huge goitre: a case report

**DOI:** 10.1186/s12893-020-00787-8

**Published:** 2020-06-11

**Authors:** Sentilnathan Subramaniam, Syamim Johan, Firdaus Hayati, Chiak Yot Ng, Nornazirah Azizan, Jitt Aun Chuah, Irfan Mohamad

**Affiliations:** 1 Department of Surgery, Queen Elizabeth Hospital, Ministry of Health Malaysia, Kota Kinabalu, Sabah Malaysia; 2 Department of Surgery, Faculty of Medicine and Health Sciences, Universiti Malaysia Sabah, Kota Kinabalu, Sabah Malaysia; 3 Department of Medicine, Faculty of Medicine and Health Sciences, Universiti Malaysia Sabah, Kota Kinabalu, Sabah Malaysia; 4 Department of Pathobiology and Medical Diagnostic, Faculty of Medicine and Health Sciences, Universiti Malaysia Sabah, Kota Kinabalu, Sabah Malaysia; 5 Department of Otorhinolaryngology-Head and Neck Surgery, School of Medical Sciences, Universiti Sains Malaysia, Kota Bharu, Kelantan Malaysia

**Keywords:** Case report, Lipoma, Minor salivary gland, Parotid gland, Sialolipoma

## Abstract

**Background:**

Sialolipoma is a rare tumour which may arise from both major and minor salivary glands and has recently been described as a variant of salivary gland lipomatous lesions.

**Case presentation:**

We report a 54-year-old male who presented with a 7-year history of large right anterior neck swelling. He was clinically euthyroid and had no compressive or infiltrative symptoms. He sought medical attention due to the discomfort exerted by the weight of the mass and was keen for excision. The swelling appeared like a goitre but physical examination proved otherwise. Imaging was suggestive of a benign tumour arising from the right parapharyngeal fossa. The mass was surgically excised and was noted to be adherent to part of the submandibular gland. Histopathological examination revealed a new variant of benign adipocytic tumour of salivary gland or sialolipoma arising from the submandibular gland. Besides being the largest sialolipoma to be reported, there are also no reports of giant submandibular sialolipomas masquerading as a huge goitre in appearance.

**Conclusion:**

Submandibular sialolipomas can present in really large sizes and appear as a giant goitre. It is important to differentiate between benign lipomas from liposarcomas and tailor the management accordingly. Surgical enucleation is the preferred choice of treatment for these benign tumours with low recurrence rates.

## Background

A huge anterior neck swelling is almost always associated with goitre by a commoner especially from the endemic region. However, that is not always the case. History, clinical examination and imaging allows us to ascertain the origin of the mass. Large anterior neck swellings theoretically can arise from the skin, subcutaneous tissue, muscle and various other structures in the region. Common causes that we usually encounter are thyroglossal cyst, lymphangioma, branchial cyst, cystic hygroma, lymphadenopathy and goitres. Lipomatous lesions of the head and neck region are generally uncommon and may arise from any structure that contains fat tissue. Sialolipoma is a rare variant of lipomatous tumour occurring within the salivary gland with less than 100 cases reported globally. Histologically, it is composed of predominantly mature adipocytes intermingled with various benign salivary gland parenchyma [1]. However, the histogenesis of sialolipoma remains unknown and is postulated to be of a hamartomatous origin [2]. We report a 54-year old gentleman who presented with a long history of right anterior neck swelling which clinically appears like a giant goitre and discuss our management strategies.

## Case presentation

**Fig. 1:**
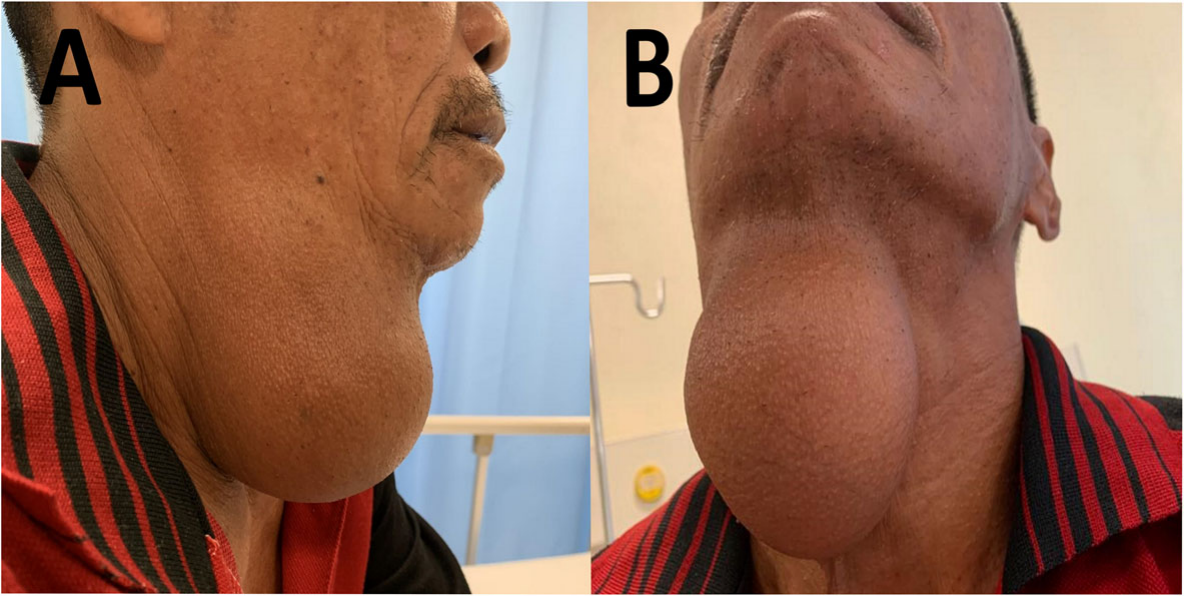
A large swelling at the right anterior triangle of the neck measuring 10 × 10 cm in size visualized from the lateral view (**a**) and anterior view (**b**)

**Fig. 2:**
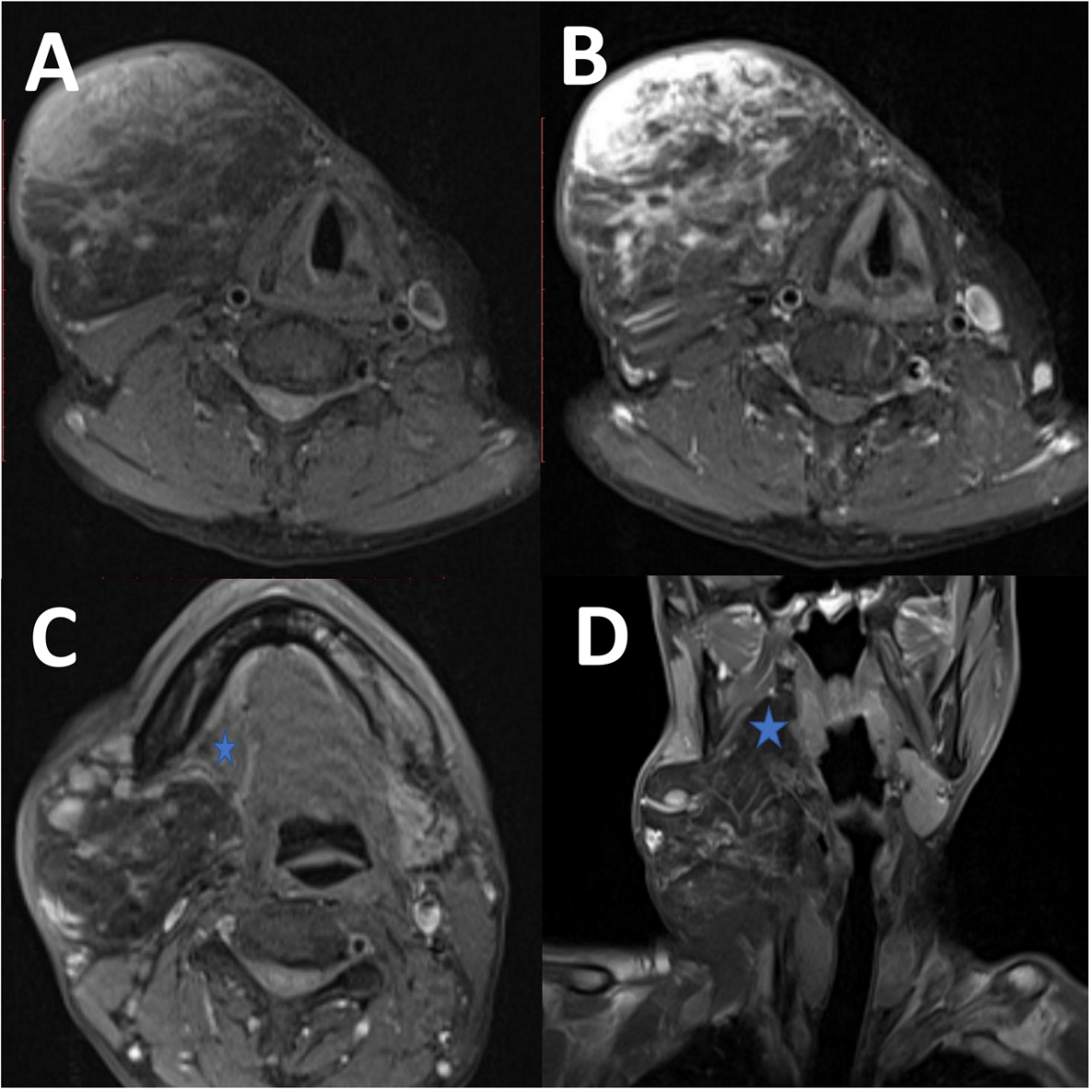
**a** Axial MRI on T1-weighted fat suppression sequence showing hyperintense soft tissue component in the anterior portion of the mass with nodular irregular septation within the mass. **b** Post contrast axial MRI on T1-weighted fat suppression sequence showing avidly enhancing soft tissue component in the anterior portion of the mass with enhancing nodular irregular septation within the mass. **c** Axial MRI on T1-weighted fat suppression sequence showing hyperintense soft tissue component in the anterior portion of the mass with nodular irregular septation within the mass. The right submandibular gland (blue star) is being displaced anteriorly by the mass. **d** Coronal MRI on T1-weighted fat suppression sequence showing the mass insinuating into the right parapharyngeal space from the right margin of the right anterior neck, elevating the right pterygoid muscles (blue star)

A 54-year-old gentleman presented with a 7-year history of right neck swelling which had progressively increased in size without compressive or infiltrative symptoms. He complained of a pulling discomfort exerted by the weight of the mass, accentuated by gravity in the erect position. He denied history of malignancy in the family or exposure to radiation. On examination, he was clinically euthyroid. The 10 × 10 cm swelling was located at the right anterior triangle of the neck extending from the submandibular region to the level of the cricoid cartilage and medially not crossing the midline (Fig. 1). The swelling was non-tender, soft in consistency and had a smooth lobulated surface. It did not move with deglutition or tongue protrusion and there were no changes to the overlying skin. Biochemical thyroid function was within normal limits. Ultrasonography revealed an ill-defined, homogenous, hyperechoic mass arising from the right submandibular space. Magnetic resonance imaging (MRI) showed a atypical lipomatous tumour with a deep extension into the right parapharyngeal space (Fig. 2). A fine needle aspiration cytology (FNAC) was performed but results were inconclusive. Therefore, we decided to proceed with surgical excision without further attempts to obtain preoperative biopsy in order to reduce the risk of tumour seeding along the biopsy tract and also because the lesion looked well encapsulated on MRI except at the right parapharyngeal fossa region.

**Fig. 3:**
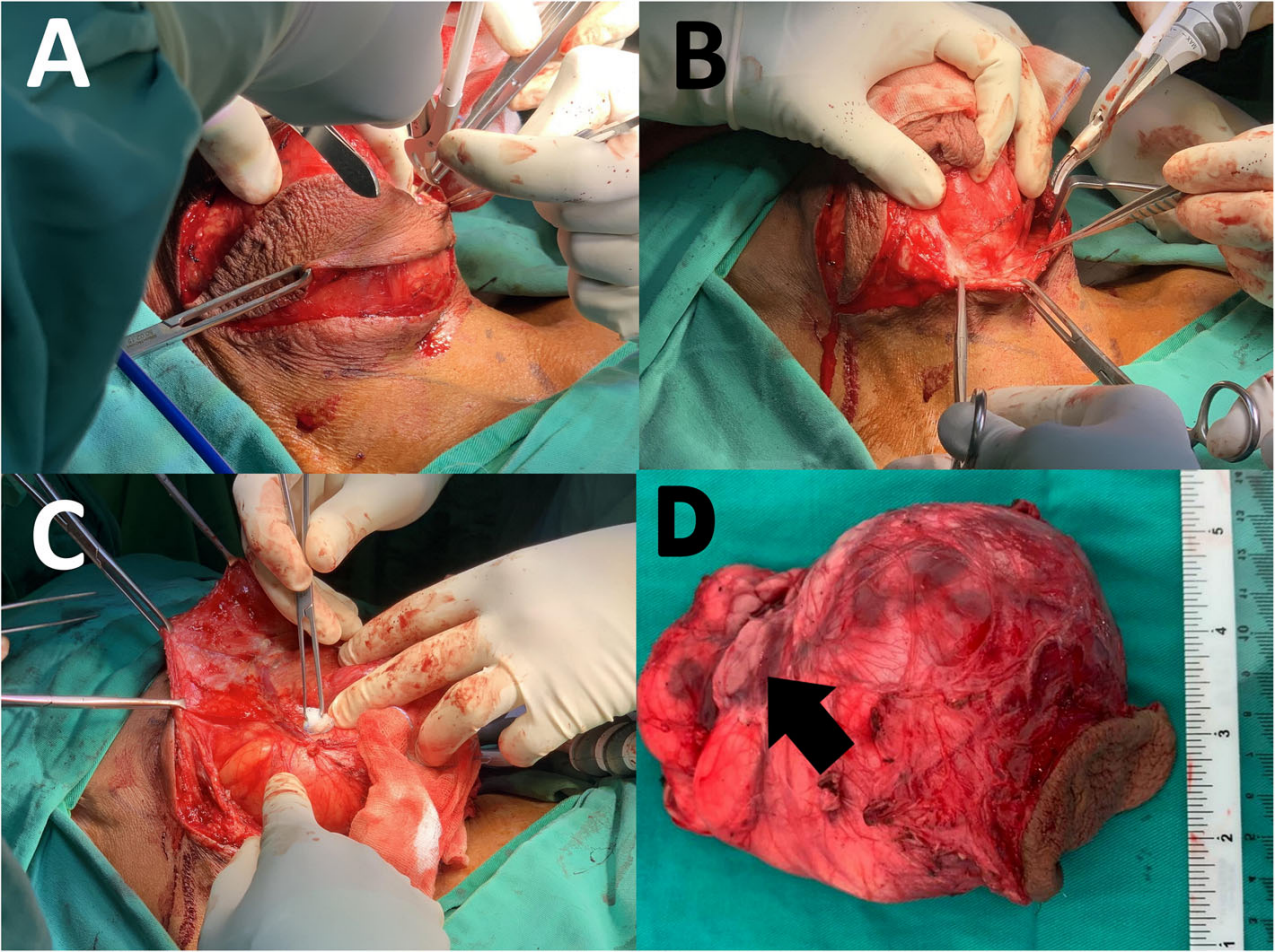
**a** Elliptical incision made and a portion of skin containing the previous FNAC tract was removed en-bloc with the tumour. This portion of skin is used to manipulate the tumor without handling the tumor itself besides providing better cosmesis by reducing excess skin. **b** Enucleation of the lesion with its capsule by creating a lower flap. An ultrasonic dissection device was used to seal and divide the peritumoral vessels and achieve haemostasis. **c** Creation of an upper flap and dissection of the engorged peritumoral vessels is demonstrated here. **d** Macroscopic appearance of the lesion showing a lipomatous tumour with intact capsule measuring 10 × 12 cm in size. The right submandibular gland was excised en bloc (black arrow)

A multidisciplinary team discussion between general surgery, radiology and otorhinolaryngology team was undertaken to discuss the MRI images and possible approaches to excise this lesion. The patient subsequently underwent excision of the lesion with the otorhinolaryngology team on standby for radical surgery if findings were suggestive of malignancy. Intraoperatively, a single, lobulated, lipomatous tumour measuring 10 × 12 cm in size with multiple engorged vessels on its surface was found to be located at the right parapharyngeal fossa (Fig. 3a - c). It was well-encapsulated with minimal adhesions to surrounding structures except at the superior part which was densely adhered to a part of the right submandibular gland. The right submandibular gland was excised en-bloc as it was most likely the origin of the tumour (Fig. 3d). There were no enlarged loco-regional lymph nodes to suggest malignancy. A small calibre vacuum drain was placed at the submandibular fossa to prevent sialocoele for a day before removal. The patient had an uneventful recovery and was discharged home on day 2 with no complications upon follow up.

**Fig. 4:**
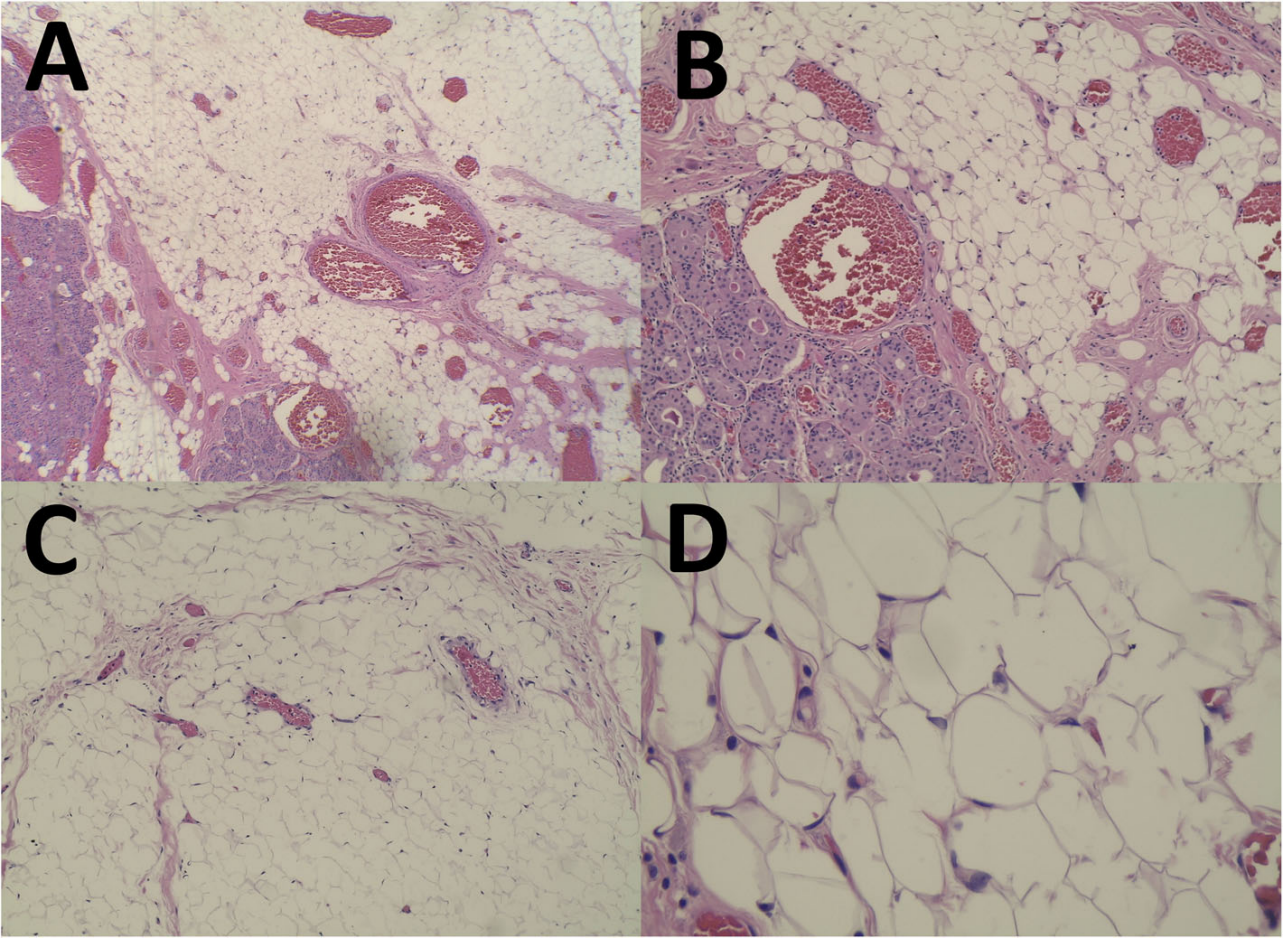
Microscopic features of sialolipoma showing a presence of lobules of mature adipose tissue separated by fibrous septae (**a** to **d**) with a presence of salivary glands acini (**a** & **b**) and higher magnification showing mature univacuolated adipocytes with no lipoblast (**d**)

Histopathological examination revealed a fairly circumscribed lesion composed of lobules of various sizes and mature univacuolated adipocytes separated by fibrous septae (Fig. 4). It measured 9.5 × 12.5 × 8.5 cm. There were multiple congested blood vessels of varying sizes and nodules composed of benign salivary gland ducts and acini with foci of squamous metaplasia. No fibrin thrombi were seen. An unremarkable salivary gland tissue was identified with no microscopic evidence of malignancy. No atypical stromal cells or lipoblasts were seen. These features were consistent with a benign adipocytic tumour of sialolipoma variant. During follow up at 1 month post-operatively, he was well without any evidence of complications or recurrence.

## Discussion and conclusion

Sialolipoma is classified as a new histological variant of lipoma instantiated by a benign salivary gland including salivary ducts and serous acini admixed with mature adipose tissues. It was first introduced by Nagao et al. before the term sialolipoma was accepted by the World Health Organization Classification of Head and Neck Tumours in 2005 [3]. It is described as a well-circumscribed and encapsulated lipomatous tumour, microscopically formed by mature adipocytes separated by fibrous septae forming lobular manner [4]. It can arise from either major or minor salivary glands in which the parotid gland is the most commonly involved organ [4]. Both males and females have an equal gender distribution with male and female preponderance to major and minor salivary glands respectively [5]. Age of occurrence ranges from 6 weeks to 74 years, with a mean of 39.4 years [4]. Tumour size ranged between 10 to 90 mm (mean: 46.2 mm) [4]. In our case, the sialolipoma arises from the submandibular gland and it is the largest of its kind to be reported till date.

The aetiology of anterior neck swellings can be divided by onset or duration, namely, congenital, acute, subacute and chronic. In this case, a 7-year history of a progressively growing neck swelling is suggestive of its chronicity. The initial primary diagnosis for these swellings can be of a thyroid pathology, laryngocele, thyroglossal duct cyst, lipoma, liposarcoma or even parathyroid carcinoma [6]. However, given such diagnoses, it is very rare for a submandibular sialolipoma to appear like a large goitre as depicted in our case. Undoubtedly, history taking and physical examination are of utmost importance in order to clinch the diagnosis.

Imaging modalities in lipomatous tumours provide a preoperative diagnosis and allows the surgical team to plan the operation besides looking for features to suggest malignancy. Simple ultrasonography can be useful but its findings vary depending on the expertise of the operator. In good hands, it can predict the tumour origin as in our case. Computed tomography (CT) and MRI are more accurate in depicting the tumour texture, location and compressive or infiltrative features compared to ultrasonography. Additionally, MRI allows us to differentiate between lipoma and liposarcoma by looking at the intensity of the fat signal and is superior to the conventional CT in this aspect. MRI findings suggestive of liposarcoma are namely thickened or nodular septa (typically thicker than 2 mm), its association with non-adipose masses and prominent foci of high T2 signal and prominent areas of enhancement [7]. Fine needle aspiration cytology is useful as the initial first-line procedure to diagnose salivary gland lesions however its accuracy is less than 50% in lipomatous tumours [8]. This reduced accuracy is due to various other lesions of the salivary gland which may contain a significant amount of adipose tissue such as lipomatous pleomorphic adenoma, lipomatosis and lipoadenoma [8]. Presence of a fibrous capsule in the histology of sialolipoma helps in distinguishing it from the others.

The treatment of choice in managing sialolipomas is surgical excision of the involved salivary gland. Guided by MRI, access to the tumour can be decided and this is a very crucial step in planning the surgery. In certain cases, the lipoma may be intramuscular hence posing a greater challenge to achieve complete tumour excision. Bleeding from the muscle is a common occurrence intraoperatively and meticulous haemostasis is important in such cases. Other possible risks and complications pertaining to this surgery include injury to the surrounding vessels and nerves, namely the facial artery, marginal mandibular nerve (branch of the facial nerve), lingual nerve and hypoglossal nerve. Post-operative salivary fistula and sialocoele are complications that need to be avoided. Nevertheless, proper planning and complete surgical excision provides good surgical outcomes as the risk of recurrence is zero [9].

In conclusion, submandibular sialolipomas can present in really large sizes and appear as a giant goitre. The attending surgeon should be able to differentiate between benign lipomatous tumours and liposarcomas through history, physical examination and imaging. Complete surgical enucleation with the involved salivary gland is the mainstay of treatment with low recurrence rates.

## Data Availability

Not applicable.

## Abbreviations

MRI: Magnetic resonance imaging
FNAC: Fine needle aspiration cytology
CT: Computed tomography
